# Metabolomic and Proteomic Identification of Serum Exosome for Hypoxic Preconditioning Participants

**DOI:** 10.1155/2023/5509913

**Published:** 2023-04-13

**Authors:** Fangcheng Fan, Yang Du, Lei Chen, Yuewen Chen, Zhifeng Zhong, Peng Li, Yong Cheng

**Affiliations:** ^1^NHC Key Laboratory of Birth Defect Research, Prevention, and Treatment (Hunan Provincial Maternal and Child Health-Care Hospital), Changsha, Hunan, China; ^2^Key Laboratory of Ethnomedicine of Ministry of Education, Center on Translational Neuroscience, School of Pharmacy, Minzu University of China, Beijing, China; ^3^Chinese Academy of Sciences Key Laboratory of Brain Connectome and Manipulation, Shenzhen Key Laboratory of Translational Research for Brain Diseases, The Brain Cognition and Brain Disease Institute, Shenzhen Institute of Advanced Technology, Chinese Academy of Sciences, Shenzhen–Hong Kong Institute of Brain Science–Shenzhen Fundamental Research Institutions, Shenzhen 518055, China; ^4^Shenzhen College of Advanced Technology, University of Chinese Academy of Sciences, Beijing 100049, China; ^5^Department of High Altitude Operational Medicine, College of High Altitude Military Medicine, Army Medical University (Third Military Medical University), Chongqing, China; ^6^College of Life and Environmental Sciences, Minzu University of China, Beijing, China

## Abstract

**Background:**

In high-altitude areas, hypoxic stress can elicit a series of physiological responses in humans. Exosomes play important roles in both local and distal cellular communications.

**Methods:**

We used ultraperformance liquid chromatography-tandem mass spectrometry (UPLC-MS/MS) and liquid chromatography-tandem mass spectrometry (LC-MS/MS) studies to analyze the differentially expressed metabolomics and proteomics in serum exosome of hypoxic preconditioning participants and control subjects in the hypoxic conditions.

**Results:**

Fifty-seven military personnel were divided into hypoxic preconditioning group (*n* = 27) and control group (*n* = 30). One hundred thirty-six differentially expressed serum exosomal metabolites were found between the hypoxic preconditioning and control groups in the hypoxic conditions, and these differentially expressed metabolites were enriched in pathways related to lysine degradation, butanoate metabolism, GABAergic synapse, histidine metabolism, and linoleic acid metabolism. In addition, hypoxic preconditioning participants showed 102 excellent differential expressions of proteomics compared to controls, which involved actin cytoskeleton organization, hemostasis, complement and coagulation cascades, vesicle-medicated transport, wound healing, etc.

**Conclusions:**

We revealed that the expression of exosomal metabolites and proteomics in hypoxic preconditioning participants was significantly different compared to controls in hypoxic conditions.

## 1. Introduction

High altitude is always accompanied by some challenges for humans native to lowlands, such as low oxygen partial pressure, cold, high energy consumption, exercise, and metabolic adjustment [1]. The main stressor at high altitudes is hypoxic stress, which affects the oxygen cascade and leads to tissue hypoxia. As oxygen is a key component of aerobic life, the main problem encountered by humans at high altitudes is the decrease of oxygen availability, leading to metabolic changes [2, 3].

In high-altitude areas, hypobaric hypoxia can elicit a series of physiological responses that are variable in humans. These responses contribute to adapting to high-altitude conditions but may lead to acute mountain sicknesses (AMS) such as high-altitude brain edema and pulmonary edema [4]. Severe AMS symptoms may temporarily debilitate. These effects may be an unpleasant nuisance for the sojourner, but for military personnel, it affects occupational performance. Therefore, it is critical for military personnel to carry out high-altitude hypoxia preconditioning, and clinical or routine laboratory tests are necessary to improve adaptability. The severity and occurrence of AMS may be driven by functional polymorphisms of key enzymes that are involved in physiological pathways, and metabolite outputs by these pathways can be determined by invoking metabolomics that is based on methods [5].

Exosomes have important roles in cell-to-cell communication, which have been explored for early-stage diagnostics, monitoring of disease status, and drug delivery [6]. In recent years, exosomes have gained more attention in the regulation of diseases on metabolome and proteome characterization [7]. Metabolomics and proteomics have been widely used to study complex systems [8, 9]. The metabolite spectrum that is generated is considered to be an effective indicator of biological physiology and assesses the interaction among a variety of proteins, genes, and the environment [10]. Therefore, applying metabolomics and proteomics to examine the physiological alterations that are caused by altitude adaptation can identify the biomarkers of AMS and further perception into the physiological pathways that affect AMS. In this study, we performed ultraperformance liquid chromatography-tandem mass spectrometry (UPLC-MS/MS) and liquid chromatography-tandem mass spectrometry (LC-MS/MS) studies to analyze the serum exosome metabolomic and proteomic profile of hypoxic preconditioning participants and control subjects in the hypoxic conditions.

## 2. Materials and Methods

### 2.1. Participants

There are fifty-seven male military personnel from a Chinese military force. Participants were divided into hypoxic preconditioning group (*n* = 27) and control group (*n* = 30). The demographic characteristics of the study subjects are shown in Table 1. All the participants have signed written informed consent. The study protocol was conducted and approved by the ethics review board, Minzu University of China. All experiments were conducted according to the Declaration of Helsinki.

### 2.2. Protocol for Hypoxic Preconditioning Paradigm

A low-pressure chamber was used to simulate altitude exposure. The training phase consists of starting from 4000 meters and rising 200 meters per day for five consecutive days (1 h for rising and fall time; target altitude time was 2 h). The control group was not trained. On the third day after the training, the hypoxic preconditioning group and control group were exposed to a simulated 4500-meter low-pressure chamber. The chamber evaluation is 8 hours in total (from 9 : 00 a.m. to 5 : 00 p.m.), excluding the rise and fall time for 1 hour, and the target altitude time was 7 hours. The evaluations of altitude reactions were collected during the whole period of target altitude. Other physiological indicators were generally analyzed 3-5 hours after being exposed to the low-pressure chamber.

### 2.3. Exosome Isolation and Quantification

A qEV column was used to isolate the exosomes as previously mentioned [11]. Briefly, serum samples from participants were collected, and exosomes were obtained on qEV method. A NanoSight system (NanoSight, London, UK) was used to measure the exosome size distribution utilizing nanoparticle tracking analysis.

### 2.4. Metabolite Measurements

As previously mentioned, serum exosome samples were subjected to a broadly focused metabolomic study employing an UPLC-MS/MS [12]. The metabolomic data management environment and the public database of metabolite information were used to implement the qualitative analysis of first-order and second-order mass spectra. The measurement of metabolites was carried out using triple quadrupole mass spectrometry and multireaction monitoring.

### 2.5. Proteome Measurements

The LC-MS/MS data-independent acquisition method was used to targeted proteome. Quantification was performed using Skyline and UniProt protein databases as described previously [13].

### 2.6. Gene Ontology Function Database

A significant bioinformatics initiative called Gene Ontology (Go) is aimed at standardizing the expression of protein across all species, including biological activities, molecular processes, and cellular components. The functional implications of the differentially expressed proteins were evaluated using the previously established Metascape enrichment approach [11].

### 2.7. Kyoto Encyclopedia of Genes and Genomes Database

The hub metabolites and differentially expressed metabolites in the module were systematically investigated for probable biological activities using MetaboAnalyst software's analysis of the Kyoto Encyclopedia of Genes and Genomes (KEGG) pathway database [14]. *P* < 0.05 was used to identify an enrichment pathway as significant.

### 2.8. Analysis of Weighted Gene Coexpression Network

For the investigation of metabolite coexpression, the R software package weighted gene coexpression network analysis (WGCNA) was used [15]. This software was employed in the current investigation to carry out coexpression network analysis. Using Pearson's correlation, we assessed the relationship between the metabolites. Additionally, we selected soft threshold capability by the near scale-free topology criteria. Through the use of the cutree dynamic function, we assigned metabolites to modules and created a signed coexpression network. Then, within each module, potential hub nodes or very important intramodule metabolites were found. With a corrected *P* < 0.05, the Benjamin-Hochberg published results for the modular trait that was significant.

### 2.9. Statistical Analysis

To determine outliers, we used principal components analysis (PCA) by the statistical function prcomp in R. Through the orthogonal partial least squares-discriminant analysis (OPLS-DA) model, which was utilized to extract the variable importance in projection (VIP) [16], differentially expressed metabolites were determined. VIP > 1.0 and *P* < 0.05 was used to identify metabolites that were differentially expressed. SIMCA software (version 14.1) was carried out for these analyses.

## 3. Results

### 3.1. Differential Expression of Serum Exosome Metabolites and Proteomics

**Figure 1:**
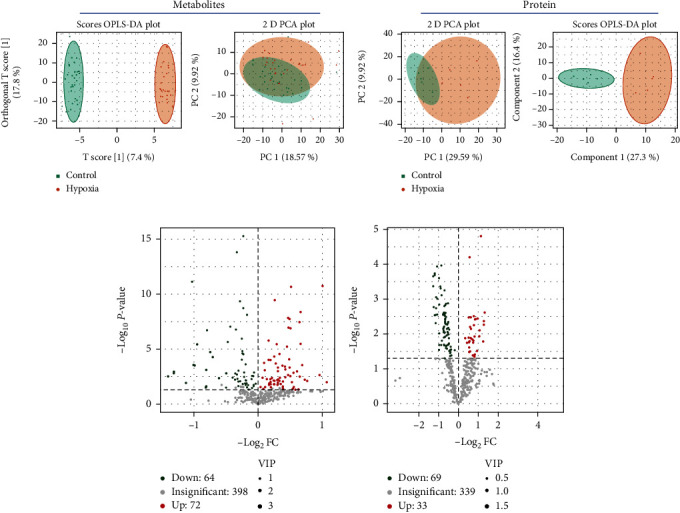
Bioinformatics screening for differential expression of serum exosome metabolites and proteomics in hypoxic preconditioning participants. (a) Principal components analysis (PCA) and orthogonal partial least squares-discriminant analysis (OPLS-DA) model plot based on the metabolites evaluated in the training participant set. (b) Principal components analysis (PCA) and orthogonal partial least squares-discriminant analysis (OPLS-DA) model plot based on the proteomics. (c) Volcano plot displaying metabolite differences between hypoxic preconditioning participants and controls for the training participant set. (d) Volcano plot displaying proteomic differences between hypoxic preconditioning participants and controls.

We used UPLC-MS/MS to analyze differentially expressed metabolites and LC-MS/MS for proteomics in serum exosomes between hypoxic preconditioning participants and controls. PCA score plots revealed distinct metabolite and proteome profiles for preconditioning participants and controls (Figures 1(a) and 1(b)). An OPLS-DA model was established to discover exosome metabolites and proteomics differently expressed between hypoxia preconditioning participants and controls (Figures 1(a) and 1(b)). For metabolites, 72 were upregulated and 64 were downregulated (Figure 1(c)). For proteomics, 33 were upregulated and 69 were downregulated (Figure 1(d)).

### 3.2. Significant KEGG Pathway Enrichment

**Figure 2:**
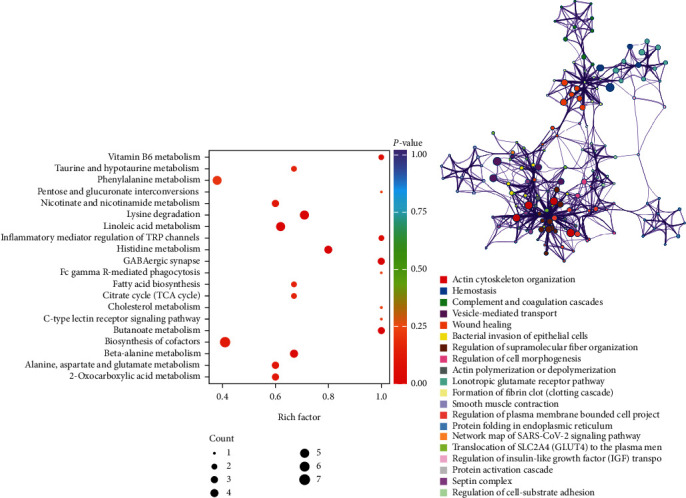
Metascape enrichment network analysis for the intra- and intercluster correlations of the (a) metabolites and (b) proteomics of the enriched clusters.

Thirteen metabolic pathways were found to be significantly affected in the hypoxic preconditioning participants compared with the controls in which lysine degradation, butanoate metabolism, GABAergic synapse, histidine metabolism, and linoleic acid metabolism were highly affected (Figures 2(a)). To evaluate the intra- and intercluster correlations of the enriched clusters, enrichment networks were created using the Metascape method.  Figure 2(b) demonstrates that the proteomic functions involved actin cytoskeleton organization, hemostasis, complement and coagulation cascades, vesicle-medicated transport, wound healing, etc.

### 3.3. Perturbation of Blood Exosomal Coexpression Modules

**Figure 3:**
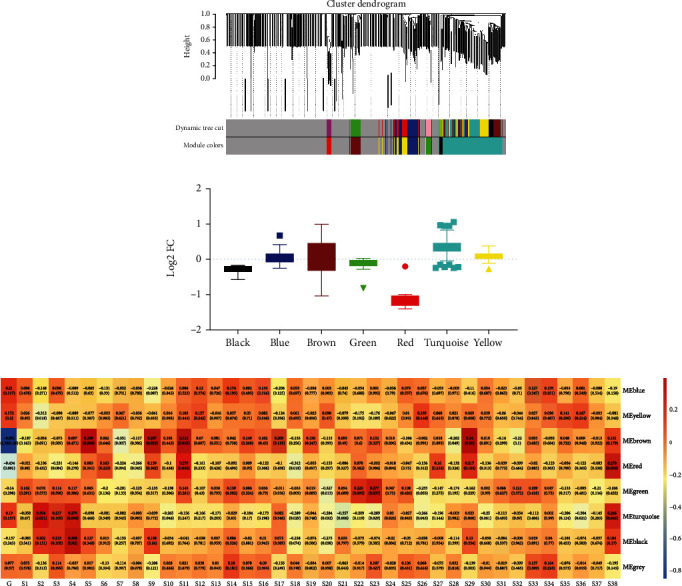
The coexpression module of serum exosome metabolites is dysregulated in hypoxic preconditioning participants. (a) Dendrogram of metabolite coexpression modules. (b) Log2 (fold change) distribution of metabolites in the modules black, blue, brown, green, red, turquoise, and yellow. (c) Pearson's correlation coefficient between S1-S38 and module eigengene. S1: nation; S2: weight; S3: height; S4: pulse (plain); S5: pulse (4500 m); S6: blood oxygen saturation (SpO2) (plain); S7: blood oxygen saturation (SpO2) (4500 m); S8: systolic pressure (mmHg) (plain); S9: systolic pressure (mmHg) (4500 m); S10: diastolic pressure (mmHg) (plain); S11: diastolic pressure (mmHg) (4500 m); S12: Lake Louise Acute Mountain Sickness Scoring System (headache); S13: Lake Louise Acute Mountain Sickness Scoring System (dizziness); S14: Lake Louise Acute Mountain Sickness Scoring System (gastrointestinal symptoms); S15: Lake Louise Acute Mountain Sickness Scoring System (fatigue and/or weakness); S16: Lake Louise Acute Mountain Sickness Scoring System (total scores); S17: left prefrontal cerebral oxygen rSO2% (plain); S18: left prefrontal cerebral oxygen rSO2% (4500 m); S19: right frontal cerebral oxygen saturation rSO2% (plain); S20: right frontal cerebral oxygen saturation rSO2% (4500 m); S21: digital decoding (plain); S22: target tracking (total average dot; plain); S23: target tracking (correct average dot; plain); S24: spatial memory (plain); S25: spatial memory (number of passes; plain); S26: manual dexterity (dominant hand; plain); S27: manual dexterity (nondominant hand; plain); S28: visual selection reaction time (plain); S29: auditory simple reaction time (plain); S30: digital decoding (4500 m); S31: target tracking (total average dot; 4500 m); S32: target tracking (correct average dot; 4500 m); S33: spatial memory (4500 m); S34: spatial memory (number of passes; 4500 m); S35: manual dexterity (dominant hand; 4500 m); S36: manual dexterity (nondominant hand; 4500 m); S37: visual selection reaction time (4500 m); S38: auditory simple reaction time (4500 m).

To further analyze the role of serum exosome metabolites dysregulation in hypoxia at the systemic level, we performed WGCNA on exosome samples from hypoxic preconditioning participants and controls to assign individual metabolites to coexpression modules and identified 8 modules (Figures 3(a) and 3(c)). In addition, the results showed that the hypoxic status was significantly correlated with four modules: two upregulated (turquoise and red) and two downregulated (black, green, and red) (Figure 3(b)). The black module represented a significant association with right frontal cerebral oxygen saturation rSO2% (4500 m), but not with the other clinical variables. The red module represented a significant association with diastolic pressure (mmHg) (4500 m) and left prefrontal cerebral oxygen rSO2% (4500 m). The green module represented a significant association with right frontal cerebral oxygen rSO2% (4500 m). The black module represented a significant association with right frontal cerebral oxygen rSO2% (4500 m).

### 3.4. Exosomal Metabolites as Biomarkers for Hypoxic Preconditioning

**Figure 4:**
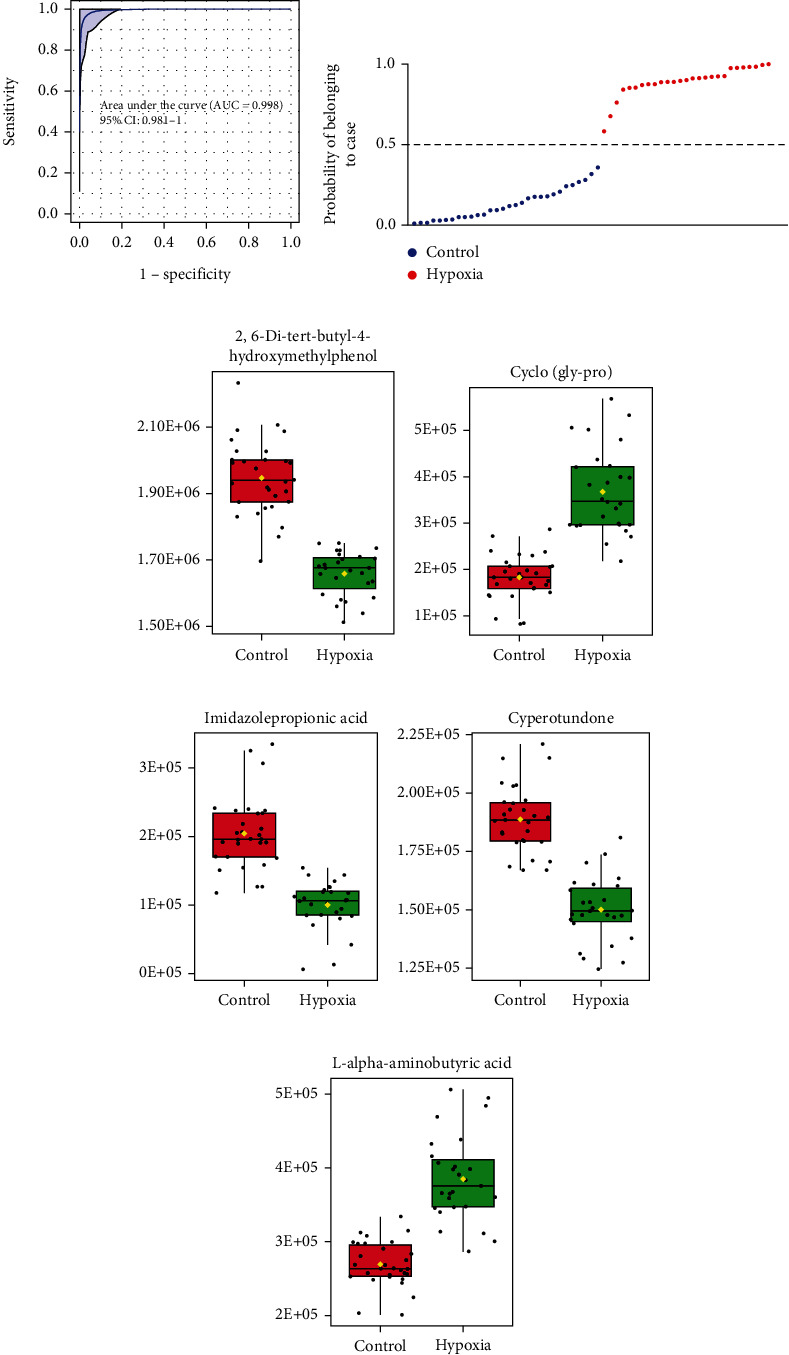
Blood exosomal metabolites as biomarkers to differentiate between hypoxic preconditioning participants and controls. (a) ROC curves were utilized to evaluate the accuracy of a cluster of 5 metabolites. (b) Scatter plot of the probability of participants belonging to cases by the 5 metabolites. Boxplot of (c) 2,6-di-tert-butyl-4-hydroxymethylphenol, (d) Cyclo (gly-pro), (e) imidazolepropionic acid, (f) cyperotundone, and (g) L-alpha-aminobutyric acid was selected. AUC: area under curve; ROC: receiver operating characteristic.

We explored whether exosomal metabolites could be served as biomarkers to differentiate between hypoxic preconditioning and the control participants. A total of 136 metabolites were subjected to potential metabolite biomarker analyses, and a set of 5 metabolites were selected as the optimal set of metabolites. We used the 5 metabolites to draw a receiver operating characteristic curve, and the area under curve (AUC) was 0.998 (95% CI, 0.98–1.0) (Figures 4(a) and 4(b)). The metabolites including 2,6-di-tert-butyl-4-hydroxymethylphenol, Cyclo (gly-pro), imidazolepropionic acid, cyperotundone, and L-alpha-aminobutyric acid were identified as the optimal set of metabolites to discriminate between hypoxic preconditioning and the control participants.

## 4. Discussion

Our metabolomic data showed a profile of differential expression of metabolites in the serum exosomes of hypoxic preconditioning participants vs. controls in hypoxic conditions. In detail, 136 differential expressions of metabolites showed significant performance in hypoxic preconditioning participants from controls. Bioinformatics analysis identified 5 metabolites which had excellent performance in distinguishing between hypoxic preconditioning participants and control subjects. In addition, hypoxic preconditioning participants showed 102 excellent differential expressions of proteomics compared with controls. Further analysis indicated the significantly enriched pathways that can be connected to lysine degradation, butanoate metabolism, GABAergic synapse, histidine metabolism, and linoleic acid metabolism. The proteomic functions involved actin cytoskeleton organization, hemostasis, complement and coagulation cascades, vesicle-medicated transport, wound healing, etc. GO function analysis showed that the function of differentially expressed proteins is mainly involved in the hypoxia-inducible factor (HIF) pathway. Taken together, data from this study showed the dysregulation of blood exosomal metabolites and proteomic contents in hypoxic preconditioning participants.

The hypoxic environment is the main stress at high altitudes, which causes blood flow into the tissue to be impaired [17]. Hypoxic hypoxia occurs when the amount of oxygen entering the blood decreases and occurs in healthy humans at high-altitude areas [18].

In addition, there are numerous stress-related signaling mechanisms, such as circulating exosomes, energy metabolism, and oxidative stress disorders [19]. The molecular response of high-altitude adaptation is complex, and serum exosomes with the function of transmitting intercellular signals may be involved. It indicates that physiological adaptation to high altitude depends on the coordination of cellular signals at the molecular level [20]. Hypoxia training is a method for adapting to high altitudes and improving physiological performance [21]. Here, we uncovered a set of metabolites and proteomics from serum exosomes and revealed differential expression of metabolites and proteomics in the serum exosomes between hypoxic preconditioning participants and controls.

Previous studies have suggested that hypoxia causes epigenetic changes in the chromatin landscape, thereby affecting the transcriptional profile of tissues [22, 23]. Exosome-mediated persistent interference between cells is believed to regulate hypoxia adaptation and rebuild the microenvironment [24]. Besides the ability to transfer ready-to-use molecules to peripheral cells, exosomes also fine-tune pathways that are necessary for cell survival during hypoxia. A recent study has suggested that hypoxia tolerance can be regulated by distant cells that have experienced hypoxia episodes and mediated by exosomes [25]. Numerous mRNAs and proteins contribute to hypoxia tolerance, and their incorporation into exosomes indicates that active HIF pathways can be transmitted to peripheral cells [26, 27]. HIF pathway has been extensively studied in understanding the adaptation to high altitude [28]. Hypoxia stabilizes the expression of HIF-1*α*, which controls the expression of numerous survival genes related to promoting cell adaptation to hypoxia, enhanced energy autophagy, and metabolism [17, 29].

Histone methylation and DNA methylation play a key role in controlling chromatin structure and gene expression [30]. The expression of several lysine-specific demethylases is induced by hypoxia, and the expression of most methylases is mediated by the action of HIF [31]. Furthermore, a previous study revealed that conjugated linoleic acid inhibits HIF-1*α* stabilization [32]. Another study reported that butanoate is presupposed of central gamma butyric acid (GABA), which is the main inhibitory neurotransmitter in the central nervous system. Plasma GABA is known as a marker of many metabolic and psychiatric disorders [33]. It has been reported that the expression of GABA is closely related to oxygen. The level of butanoate was increased in hypoxic infants, and the catabolic pathway of GABA produces succinic and butanoic acid, both of which were acutely increased in hypoxic animals [34]. In addition, the expression of hypoxia-induced HIF-1 in the cerebral cortex is regulated by succinic acid in the tricarbonic cycle and GABA shunt reactions [35]. Therefore, there is a connection between lysine degradation, butanoate metabolism, histidine metabolism, GABAergic synapse, and the HIF pathway. It is also applicable to our analysis results of metabolism from serum exosomes of hypoxic preconditioning participants. In this study, we found that hypoxic preconditioning participants may change the expression of series metabolites in the serum exosome to adapt to the hypoxic environment.

Hypoxia stress is commonly harmful to the organism but may be beneficial in some cases. Reduced oxygenation leads to the induction of many hypoxia-responsive proteins such as HIF pathway-related proteins [36]. In hypoxic conditions, tissue perfusion is reduced, and requirements for O_2_ availability are inadequate to meet tissue [37]. Hypoxia signaling controls vascular permeability, vascular growth, and the repair of vascular injury [38]. Numerous genes that are involved in regulating vascular homeostasis are direct or indirect targets of the HIF pathway [39]. HIF-1 controls oxygen delivery to regulate glucose metabolism and redox homeostasis, which mediates angiogenesis and vascular remodeling [40]. Briefly, HIF-1 activates the transcription of a series of target genes such as PDLIM1, TPM2, CALD1, and TPM4. These target genes encode proteins that regulate adaptive responses to hypoxia such as cytoskeleton function, erythropoiesis, and angiogenesis [41, 42]. In addition, hypoxic preconditioning significantly increased the wound healing potential. It enhances wound angiogenesis and promotes epithelial regeneration in the early stage of wound healing [43]. In this study, our study demonstrates that the primary functions involved in hypoxia preconditioning included hemostasis, complement, coagulation cascades, and wound healing, which suggests the potential role of tissue hypoxia preconditioning. Vascular remodeling involves the reorganization of the actin cytoskeleton [44]. The cytoskeleton contributes to cell shape, cell division, and maintenance of multicellular tissue, including the maintenance of the epithelial barrier [45]. In endothelial cells after hypoxia, it causes a profound reorganization of the actin cytoskeleton [46]. The hypoxia-induced impaired cytoskeleton organization leads to the functional changes of vascular remodeling. Hypoxia damages the cytoskeleton organization and then adversely affects the transport of ions and liquids [47]. A previous study has shown that hypoxia can increase the release of many inflammatory cytokines that regulates drug metabolism. In addition, hypoxia-inducible HIF-1 and microRNA-regulated pathways play a critical role in mediating medicated transport [48]. Therefore, after hypoxia preconditioning, the corresponding protein such as HIF pathway-related proteins is achieved which will be adapted to the hypoxia environment [49]. Our results suggested that hypoxia preconditioning leads to the differential expression of cytoskeleton reorganization, hemostasis, complement and coagulation cascades, vesicle-medicated transport, wound healing, etc. in serum exosome proteomics compared to nonhypoxia preconditioning participants in hypoxic conditions.

It has been reported that hypoxic stress would induce cytotoxicity, inflammation, and oxidative stress [50]. In this study, five potential metabolite biomarkers that separated hypoxic preconditioning from control individuals were identified. Among them, Cyclo (gly-pro) and L-alpha-aminobutyric acid were upregulated and 2,6-di-tert-butyl-4-hydroxymethylphenol, imidazolepropionic acid, and cyperotundone were downregulated when participants in hypoxic preconditioning condition. These metabolites are closely associated with antinociceptive, anti-inflammatory effects. 2,6-Di-tert-butyl-4-hydroxymethylphenol is reported as an antioxidant that inhibits rTNF-alpha-induced cytotoxicity [51]; Cyclo (gly-pro) is indicated to have antinociceptive and anti-inflammatory effects [52]. Imidazolepropionic acid is closely related to the regulation of blood pressure [53]. Cyperotundone has antinociceptive, anti-inflammatory, and redox properties [54]. L-Alpha-aminobutyric acid was upregulated in autoimmune diseases [55]. Given that several highly differentially expressed proteins and metabolites in participants under hypoxia preconditioning, the functions of these molecules are mainly involved in anti-inflammation and antioxidation, and therefore, it is possible that these molecules could serve as targets to inform the AMS susceptibility. However, to determine whether a person is AMS susceptible or not by the serum exosomal proteins and/or metabolites requires a complete different study design, which should recruit volunteers with and without development of AMS for assessing our discovered serum exosomal proteins and/or metabolites, ideally before and after exposed to plateau, to validate the potential biomarkers. Therefore, a limitation of this study is that the usefulness of these potential biomarkers to inform AMS susceptibility is unclear, and it warrants further well-designed study to address the issue.

In conclusion, we found that the expression of metabolites and proteomics of blood exosomes in hypoxic preconditioning participants were significantly different compared to controls in hypoxic conditions. The main enriched metabolites terms are connected to lysine degradation, butanoate metabolism, GABAergic synapse, histidine metabolism, and linoleic acid metabolism. Moreover, the proteomic functions involved actin cytoskeleton organization, hemostasis, complement and coagulation cascades, vesicle-medicated transport, wound healing, etc.

## Figures and Tables

**Table 1:** Demographic characteristics of the subjects.

Group	Control group	Hypoxic preconditioning group
*n*	30	27
Age	22.67 ± 1.83	21.96 ± 1.65
Weight	67.87 ± 7.76	68.78 ± 7.44
Height	173.4 ± 4.48	173.7 ± 5.17

## Data Availability

The data used to support the findings of this study are available from the corresponding author upon request.
